# Antibody-Loading of Biological Nanocarrier Vesicles Derived from Red-Blood-Cell Membranes

**DOI:** 10.1021/acsomega.4c00650

**Published:** 2024-05-14

**Authors:** Maryam Sanaee, K. Göran Ronquist, Elin Sandberg, Jane M. Morrell, Jerker Widengren, Katia Gallo

**Affiliations:** † Department of Applied Physics, School of Engineering Sciences, KTH Royal Institute of Technology, Stockholm 10691, Sweden; ‡ Department of Clinical Sciences, Swedish University of Agricultural Sciences, Uppsala 75007, Sweden

## Abstract

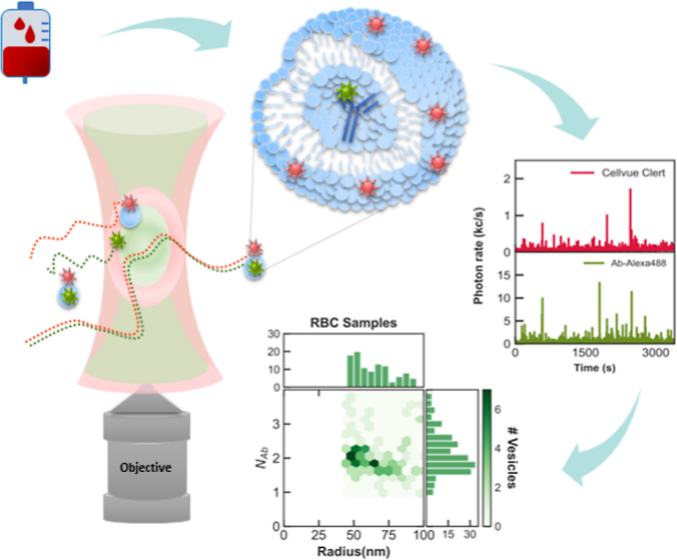

Antibodies, disruptive potent therapeutic agents against pharmacological targets, face a barrier in crossing immune systems and cellular membranes. To overcome these, various strategies have been explored including shuttling via liposomes or biocamouflaged nanoparticles. Here, we demonstrate the feasibility of loading antibodies into exosome-mimetic nanovesicles derived from human red-blood-cell membranes, which can act as nanocarriers for intracellular delivery. Goat-antichicken antibodies are loaded into erythrocyte-derived nanovesicles, and their loading yields are characterized and compared with smaller dUTP-cargo molecules. Applying dual-color coincident fluorescence burst analyses, the loading yield of nanocarriers is rigorously profiled at the single-vesicle level, overcoming challenges due to size-heterogeneity and demonstrating a maximum antibody-loading yield of 38–41% at the optimal vesicle radius of 52 nm. The achieved average loading yields, amounting to 14% across the entire nanovesicle population, with more than two antibodies per loaded vesicle, are fully comparable to those obtained for the much smaller dUTP molecules loaded in the nanovesicles after additional exosome-spin-column purification. The results suggest a promising new avenue for therapeutic delivery of antibodies, potentially encompassing also intracellular targets and suitable for large-scale pharmacological applications, which relies on the exosome-mimetic properties, biocompatibility, and low-immunogenicity of bioengineered nanocarriers synthesized from human erythrocyte membranes.

## 1 Introduction

Advances in production of high-affinity antibodies (Ab) harnessed with dedicated pharmacological actions are paving the way for targeting previously untreatable diseases^1−6^ and hold significant potential for the development of novel immunotherapeutic agents boosting the effectiveness of tumor treatments besides chemotherapy.^1,7,8^ However, most of the available methods for antibody delivery are restricted to extracellular or cell-surface-bound targets,^1−11^ and there is still huge demand to develop other important class of antibodies against intracellular targets.^8,10−13^ Major difficulties in Ab-deployment against intracellular targets stem from their relatively large size and chemical composition, preventing them from naturally crossing the cell membranes and limiting their blood-circulation times and therapeutic action in the absence of appropriate protective encapsulation.^5,6,9−14^

Overcoming these challenges is crucial to establishing Ab therapies within intracellular spaces. Accordingly, significant research efforts are being devoted to devising efficient methodologies for the delivery of antibodies across the immune system and cell membranes, ranging from intracellular injection to camouflaged transport techniques.^14^ The former relies on harsh mechanical disruption of the cell membrane through injection or electroporation, with limited loading efficiency and significant impact on cell viability, exclusively suitable for in vitro studies.^15,16^ Alternative approaches involve antibody camouflaging using cell-penetrating peptides, engineered nanoparticles, or liposomes to facilitate antibody transport across cellular membranes.^5,6,9−13,17,18^ Among these, nanocarrier-assisted delivery, employing polymeric nanoparticles,^5,19^ lipid nanovesicles,^20^ and nanoparticles camouflaged with the aid of biomimetic coatings derived from cell membranes,^21−25^ stands out as a promising approach for drug delivery. Liposomes, known for biocompatibility and controlled release properties, face limitations due to protein corona formation and short-term cargo preservation effects.^26^ Some challenges can be mitigated by PEG-polymerization,^19^ which, however, may trigger anti-PEG immunoglobulin production in vivo, resulting in lowered blood circulation times and degraded immunogenicity.^27,28^

The innate biocompatibility and nonimmunogenicity of red blood cell (RBC) membranes make them ideal raw materials for direct use as biocamouflaging materials in a variety of treatments and as drug carriers for intracellular delivery in nanovesicle forms.^24,29,30^ RBC membrane-coated nanocarriers have been already studied for Ab delivery and proven to afford longer circulation times thanks to functional RBC-membrane proteins such as CD47.^22,23,28,29,31^ However, the use of such RBC-camouflaged nanocarriers requires the Ab cargo to be aggregated first into a solid form,^21,25^ which may compromise its functionality and induce complications.^32^ Such drawbacks can be overcome by drug carriers directly synthesized from RBC-membranes in the form of nanovesicles, provided that suitable procedures become available for their loading with antibodies.^33,34^

Recently, a novel methodology was devised for synthesizing and loading RBC-derived nanovesicles, similar to exosomes, enabling large-scale production in stable formulations with engineerable properties. This technique, initially applied to vesicle loading with dUTP cargo molecules,^35^ is here further developed to demonstrate the loading of RBC membrane-derived nanovesicles with larger molecular cargos, specifically goat-antichicken IgY (H + L) secondary antibodies with significantly larger molecular weights (∼145 kDa) than labeled dUTP (∼1 kDa). This study systematically analyzes and quantitatively compares the results of Ab-loading with dUTP-loaded vesicles under identical processing conditions, employing spectroscopic protocols developed for single-vesicle profiling with single-molecule resolutions.^33,35^ The findings reveal that Ab-loading yields are maximized for slightly (∼5–10 nm) larger vesicle radii than the ones of dUTP-loading, consistent with the smaller size of the latter, yet still in the ∼50 nm radius range typical of exosome-mimetic nanocarriers. Additional cleaning of nanocarrier solutions using an exosome spin column shows comparable average loading yields of 14% for both Ab and dUTP. The inferred average number of cargo molecules loaded in each nanovesicle also features very similar values (2.25 for Ab and 2.49 for dUTP), exceeding two in both cases, despite their large size discrepancy. The results provide clear evidence of the viability of human erythrocyte-derived nanovesicles for Ab-loading and pave the way to their exploitation as a novel biomimetic system for potential antibody in vivo delivery.

## 2 Methods and Experiments

**Figure 1:**
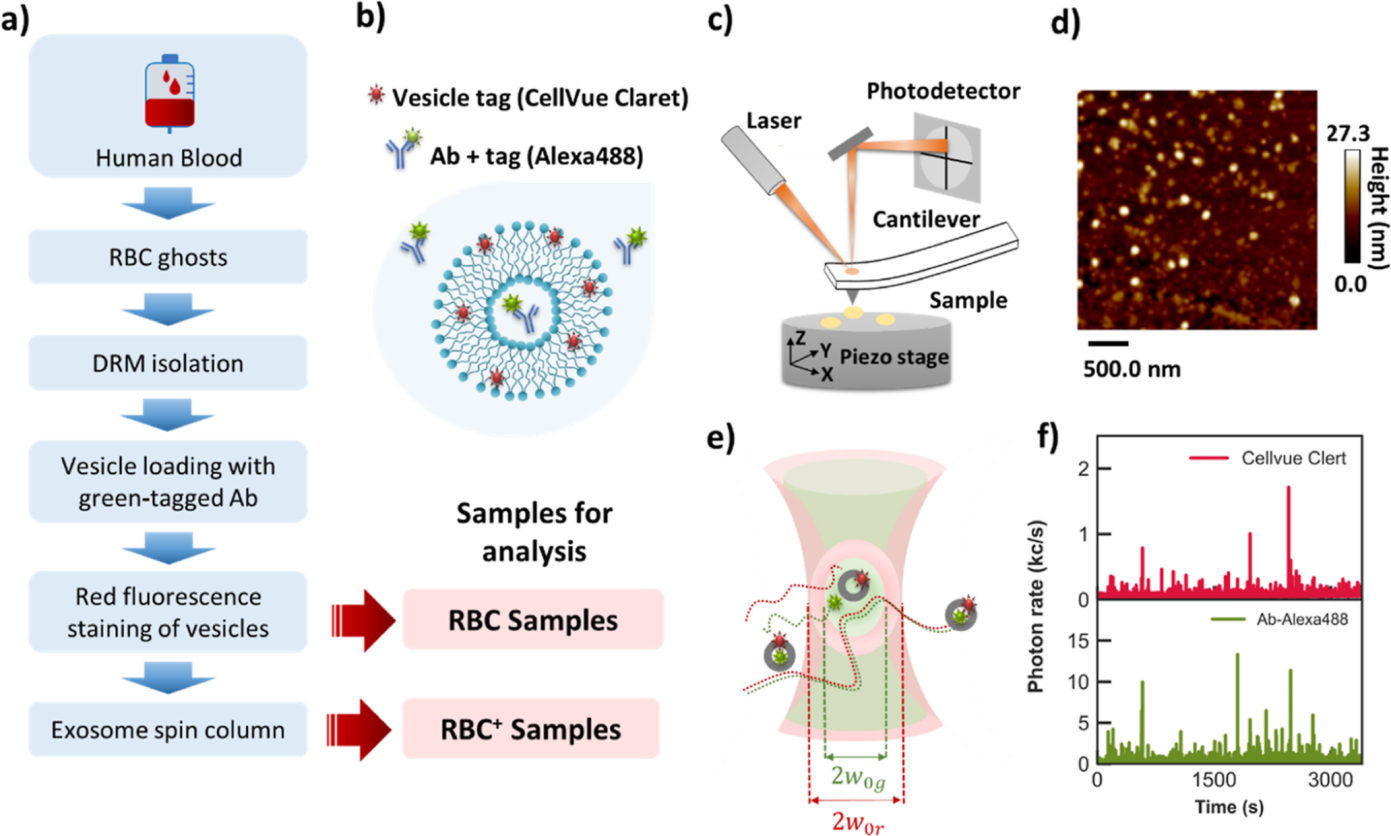
(a) Preparation steps of antibody-loaded nanovesicles. Ab = antibody, RBC = red blood cell nanovesicles, and RBC^+^ = RBC with additional solution cleaning. (b) Dual-color fluorescent staining scheme for the Ab cargo (Alexa488, green dye) and the nanovesicle (CellVue Claret, red dye). AFM: (c) experimental setup and (d) image of nanovesicles. DCFM: (e) setup and (f) typical time traces in detection for the red (vesicles) and the green (antibody molecules) signal channels. Coincident red and green temporal bursts denote Ab-loaded nanovesicles.

Figure 1a provides a flowchart for the preparation of antibody-loaded nanovesicles, closely following previously developed procedures for dUTP cargo molecules.^36^ The method involves multiple ultracentrifugation steps to purify RBC ghosts from human blood^37^ and isolate exosome-like vesicles from detergent-resistant membrane (DRM) solutions at a buoyancy of 30% sucrose (1.13 mg/cm^3^) in sucrose gradients (details in Supporting Information). Ab-loading is performed through posthypertonic lysis of RBC vesicles,^38^ inducing vesicle rupture and their subsequent revesiculation, upon which they may engulf Ab-molecules deliberately dispersed in physiologic buffer (see also S1). For this pilot study, a goat-antichicken IgY (H + L) antibody is chosen as the cargo molecule. The antibody is conjugated with AlexaFluor488 (Thermo Fisher) for green fluorescence tagging in optical characterizations using dual-color fluorescence microscopy (DCFM). The outer membranes of the nanovesicles are further stained with CellVue Claret (Sigma-Aldrich) for far-red fluorescence. The dual-color green and red tagging scheme is illustrated in Figure 1b. As highlighted in Figure 1a, the sample preparation process encompassed two slightly different sample typologies of nanovesicles, denoted as RBC and RBC^+^. The main difference between the two consists in an additional cleaning step performed at the end on the latter (RBC^+^), with an exosome spin column purification procedure,^39^ as detailed in S1. In all cases, loaded and tagged RBC or RBC^+^ nanovesicles, dispersed in PBS solution, underwent systematic characterizations by means of atomic force microscopy (AFM, Figure 1c,d) and confocal fluorescence microscopy (Figure 1e,f), according to experimental and analytical protocols originally defined in previous publications.^35,40^

The considerable heterogeneity of the exosome-mimetic nanovesicles under investigation necessitates characterizations at the single-vesicle level to extract critical physical parameters, such as size distribution, and facilitate thorough assessments of antibody loading. To address these challenges, we employ experimental methodologies that involve concurrent AFM and DCFM measurements (Figure 1c–f) and dual-color coincident fluorescence burst (DC–CFB) analyses for size-resolved characterizations of both carrier nanovesicles and their cargos. We exclusively considered bursts surpassing predefined thresholds for minimum photon number (M) and count rate (F) for both red and green bursts, as outlined in the Supporting Information (S2). By these burst selection criteria, the bursts originating from particles with shorter dwell times within the detection volume, potentially caused by trajectories passing only through the peripheral region of the detection volume and lower photon counts, could be filtered out. We also systematically compared the red fluorescence results with independent AFM measurements to validate the accuracy of the extracted size distributions for whole nanovesicles.

## 3 Results and Discussion

**Figure 2:**
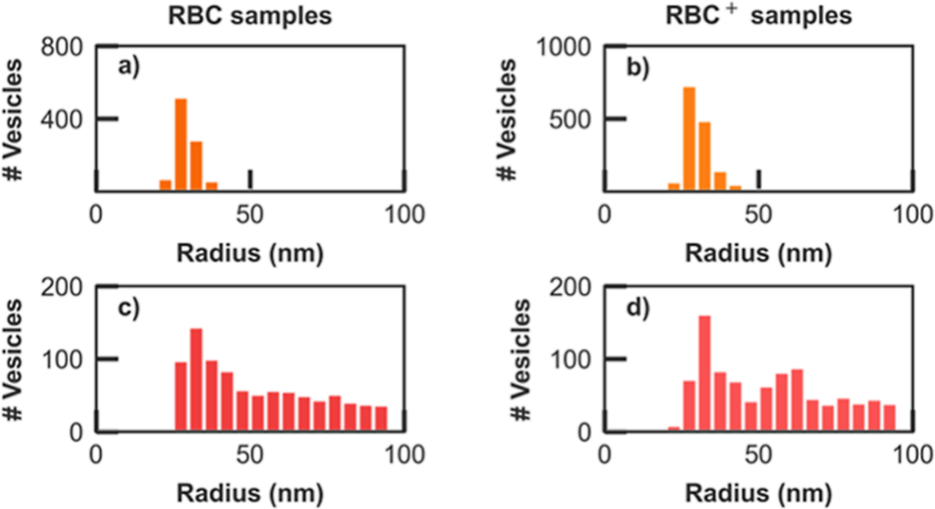
Size histograms of the whole populations of nanovesicles assessed through (a,b) AFM and (c,d) red burst analysis in fluorescence microscopy experiments, for RBC (plots a, c) and RBC^+^ (plots b, d) sample preparations. The AFM measurements were taken from dried samples rather than from a liquid medium, leading to deviations from the observed vesicle size in red fluorescence distributions, as reflected in the longer tail observed in size histograms (c, d) for larger vesicle sizes.

Figure 2 presents key outcomes of the AFM and fluorescence characterizations conducted on the overall populations of RBC and RBC^+^ nanovesicles following their synthesis and Ab-loading procedure. Detailed AFM investigations confirm the formation and integrity of single DRM nanovesicles for both sample typologies (RBC and RBC^+^), featuring size ranges and distribution profiles akin to those of exosomes and exosome-mimetic nanovesicles.^35,41−43^ As illustrated by the plots of Figure 2a,b, and further quantified by the data in Table 1, the AFM histograms for the two sample preparations appear to peak at the same vesicle radius, i.e.: *R*_max_^AFM^ = 27 nm, and exhibit comparable values of their average radius, i.e.: *R*_ave_^AFM^ = 30 nm for RBC, and 31 nm for RBC^+^ samples.

**Table 1:** Summary of the Statistics of the RBC and RBC^+^ Nanovesicle Populations Subject to the Loading Procedures of Figure 1a, with Ab or dUTP as Cargo Molecules, Retrieved from AFM and Red Fluorescence Burst Analyses^a^

	AFM		fluorescence microscopy	
sample typology	*R*_max_ (nm)	*R*_ave_ ± σ_R_ (nm)	*R*_max_ (nm)	*R*_ave_ ± σ_R_ (nm)
Ab-RBC	27	30 ± 4	32	59 ± 26
Ab-RBC^+^	27	31 ± 5	32	60 ± 26
*dUTP*-RBC	*27*	*32±8*	*27*	*49±22*
*dUTP*-RBC^+^	*27*	*30±4*	*32*	*56±25*

a *R*_max_ = nanovesicle radius at the peak of their size-distribution, *R*_ave_ = average radius for each vesicle population, and σ_R_ = standard deviation of the vesicle radius.

Complementary insights into the hydrodynamic size populations of two nanovesicles are obtained by confocal fluorescence microscopy experiments (Supporting Information) through a burst analysis of the red fluorescence signal time traces, yielding the distributions shown in Figure 2c,d, for RBC and RBC^+^ samples, respectively. For a direct comparison, Table 1 reports the values retrieved by AFM and fluorescence measurements for both preparations subject to Ab- (rows 1–2) and dUTP- (rows 3–4) loading processes. The latter (see also Supporting Information) were processed at the same time and under identical experimental conditions for a direct comparison with the Ab-loading cases and to provide a reference to previous literature.^35^ DCFM experiments, as outlined in S1, provide comprehensive insights into nanovesicle populations and their loading.^35^ The burst analysis of time traces from the red membrane dye (Figure 1b) in the fluorescence experiments (Figure 1c) allows for the retrieval of size-dependent statistics for all nanovesicle populations, as depicted in Figure 2c,d.

Fluorescence-derived estimates for the size of nanovesicles tend to be slightly larger than those obtained by AFM. This discrepancy is attributed to the larger hydrodynamic size of vesicles in physiological solution (the setting for fluorescence microscopy measurements) compared to dry conditions used for AFM. Practically, the AFM topography images were captured from dried samples rather than in a liquid medium. This distinction could result in some deviations from the vesicle size observed in red fluorescence distributions, particularly evident in the longer tail observed in size histograms of fluorescence measurements for larger vesicle sizes. Therefore, the primary value of conducting a comparative analysis of fluorescence and AFM histograms lies in qualitatively tracking the overall trends in size distributions. The observed shift in the peak radius of fluorescence and AFM distributions is relatively small (*R*_max_ – *R*_max_^AFM^ ∼ 5 nm). However, the difference becomes more pronounced when considering the average values of vesicle radii (*R*_ave_ – *R*_ave_^AFM^ ∼ 30 nm) due to the random diffusion trajectories through the detection volume and the tendency of biomimetic nanovesicles to aggregate in physiological solutions, consistent with prior reports.^35^ This effect, well documented in the literature,^44^ is confirmed by the longer tails in the distributions obtained from fluorescence data, particularly visible for *R* ≫ 30 nm in Figure 2c,d and essentially absent in the narrower AFM profiles of Figure 2a,b. Furthermore, both AFM and fluorescence results consistently indicate no significant impact of the additional cleaning step (RBC vs RBC^+^) on Ab-loaded sample preparations. The maximally populated nanovesicle radius derived from the fluorescence data analysis remains the same for both RBC and RBC^+^ samples (*R*_max_ = 32 nm) and this is equally true for their average radii (*R*_ave_ ∼ 60 nm).

A notable observation from comparing Ab and dUTP loading results is the larger vesicle size associated with antibody loading. This aligns with the substantial weight difference between Ab molecules and dUTP, with the former being over 2 orders of magnitude higher molecular weight than the latter. A distinct difference is observed in the values of *R*_max_ and *R*_ave_ after the extra cleaning procedure (RBC vs RBC^+^) applied to dUTP-loaded samples, an effect not observed in antibody loading. In the dUTP case, both *R*_max_ and *R*_ave_ show an increase of approximately 5–7 nm postcleaning. This suggests additional size-filtering effects during the exosome spin column process, potentially favoring slightly larger vesicles and better matching the size-distribution peak (*R*_max_) of Ab-loaded samples, which is approximately 5 nm larger than that in the dUTP case. The impact of cleaning (RBC vs RBC^+^) is more pronounced in the case of dUTP loading compared to Ab loading, significantly affecting also the retrieved loading yields, as discussed in the next section.

**Figure 3:**
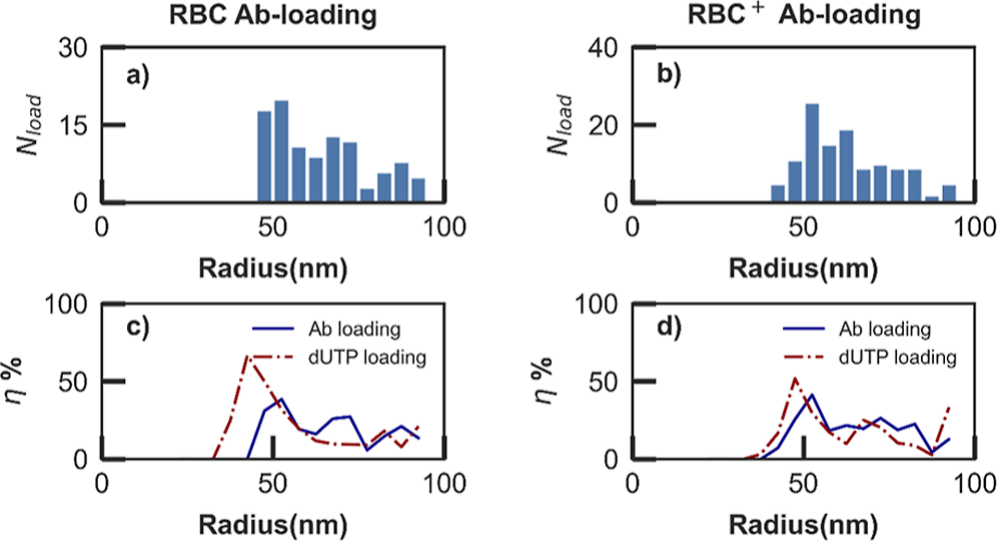
Histogram of the number of Ab-loaded nanovesicles (*N*_load_) as a function of nanovesicle radius R, for: (a) RBC and (b) RBC^+^ sample preparations. Size-distribution of the loading yield η(*R*), for Ab (solid lines) and dUTP (dashed lines) cargo molecules: (c) RBC and (d) RBC^+^ preparations.

Figure 3 illustrates the result of further investigations into the subpopulations of loaded nanovesicles performed by DC–CFB analysis, considering nanovesicles loaded with antibodies and their dUTP-loaded counterparts (Supporting Information and ref (35)). Figure 3a (b) shows the size distribution of Ab-loaded RBC (RBC^+^) nanovesicles, while Figure 3c (d) illustrates their loading yield, i.e.: , quantified as the ratio of the number of loaded nanovesicles (*N*_load_), determined from coincident green and red bursts (Figure 3a,b), and the total nanovesicle count (*N*_tot_), determined from red burst analyses (Figure 2c,d), as a function of the nanovesicle radius *R*. Table 2 summarizes key figures of merit extracted by the DC–CFB analysis of the experiments to enable quantitative comparisons between RBC and RBC^+^ preparations as well as loading with Ab and dUTP cargo molecules.

**Table 2:** Summary of the Statistics of the Loaded Sub-Populations of RBC and RBC^+^ Nanovesicles, with Ab and dUTP Cargo Molecules, Retrieved from DC–CFB Analyses^a^

	*R*_max_^load^ (nm)	<>*R*_ave_^load^ ± σ_R_<> (nm)	η_max_ (%)	*Η*_av_ (%)	*N*_max_	*N*_ave_ ± σ_N_
**Ab-RBC**	52	66 ± 15	38	14	1.75	2.25 ± 0.75
**Ab-RBC**^**+**^	52	65 ± 15	41	14	2.25	2.53 ± 0.76
*dUTP-RBC*	*42*	55 ± 17	*67*	*20*	*2.25*	*2.71±0.71*
*dUTP-RBC*^*+*^	*48*	61 ± 17	*52*	*15*	*2.25*	*2.49±0.54*

a *R*_max_^load^ = loaded nanovesicle radius at the peak of their size distribution, *R*_ave_^load^ = average radius of the loaded vesicles, and σ_R_ = standard deviation of the vesicle radius. η_max_ = loading yield for R = *R*_max_^load^ and η_ave_ = average loading yield. *N*_max_ and *N*_ave_ are the maximum and average number of loaded cargos per vesicle (*N*_Ab_ or *N*_dUTP_), respectively.

Similar to dUTP-loaded nanovesicles, the Ab-loaded nanovesicle populations exhibit a skewed distribution in radius, with a primary peak at *R*_max_^load^ < 70 nm and an extended tail toward larger sizes (>100 nm), where experimental artifacts due to vesicle agglomeration combined with random diffusion trajectories become prominent. The size distributions of Ab-loaded nanovesicles in RBC (Figure 3a) and RBC^+^ (Figure 3b) samples show essentially the same values for the peak (*R*_max_^load^ = 52 nm) and average (*R*_ave_^load^ ∼ 65 nm) radii, indicating negligible impact of the solution cleaning step. Comparing the histograms in Figure 3a,b with those in Figure 2c,d, depicting the total nanovesicle populations for the Ab-loading case, highlights a shift in the vesicle distributions toward larger sizes upon loading. This shift is quantified by comparing the values for *R*_max_ in Table 1 and *R*_max_^load^ in Table 2, revealing an increase of ∼20 nm in the peak radius of Ab-loaded compared to the whole nanovesicle populations. This size increase in the loaded nanovesicle population is also apparent in the values of the average radii of Ab-loaded (*R*_ave_^load^, Table 2) and whole (*R*_ave_, Table 1) vesicle populations. In comparison to dUTP-loaded vesicles, the Ab-loaded vesicles exhibit approximately 10 nm-larger average and peak sizes, consistent with the larger size and molecular weight (∼145 kDa) of antibodies compared to labeled dUTP molecules (∼1 kDa). Another notable difference is observed in the dUTP-loaded vesicle distributions after the extracleaning process, evident from the data in S3 and Table 2, indicating an increase by 6 nm in both *R*_max_^load^ and *R*_ave_^load^ of RBC^+^ versus RBC dUTP-loaded nanovesicles. Consistently, there is a noticeable shift toward larger sizes in the statistics of the overall nanovesicle populations when comparing RBC and RBC^+^ preparations in the case of dUTP. This emphasizes the size-filtration effect of the original nanovesicle populations associated with the RBC^+^ cleaning step, which tends to favor slightly larger vesicles (with another notable difference as they have a diameter of 50 nm or more) that better align with Ab-loaded vesicles. This clarifies the observed changes affecting the dUTP-loaded vesicles but not the Ab-loaded vesicles as well as the adjustments in the dUTP-loading yield results in RBC and RBC^+^ preparations, as shown in Figure 3c,d (dashed lines) and Table 2 (η_max_ and η_ave_ in rows 3–4).

Further analysis of the dual-color experimental data is explained in Supporting Information, enabling size-resolved evaluations of loading yields, as depicted in Figure 3c,d for RBC and RBC^+^ samples, respectively. Consistent with previous discussions, the Ab-loading yield distribution, η(*R*), remains unaffected by the cleaning procedures, peaking at the same vesicle radius, *R*_max_^load^ = 52 nm, for both RBC and RBC^+^ preparations. The maximum loading yield, η_max_ = η(*R*_max_^load^), is also minimally affected, with values of 38 and 41% for RBC and RBC^+^ samples, respectively (Table 2). Average values of loading efficiencies and carrier vesicle sizes exhibit similar trends, with η_ave_ = 14% and *R*_ave_^load^ ∼ 65 nm, respectively, regardless of the extra cleaning step in the Ab case. However, this is not observed for the dUTP case, as is evident in the loading yield distributions for RBC and RBC^+^ preparations (dashed lines in Figure 3c,d) and the corresponding figures of merit in Table 2. The RBC^+^ cleaning step induces a clear modification of the peak yield, with *R*_max_^load^ increasing from 42 to 48 nm and *R*_max_^load^ decreasing from 67 to 52%, along with substantial effects on average values, with *R*_ave_^load^ increasing from 55 to 65 nm and η_ave_ decreasing from 20 to 15%. These trends align with those highlighted in the vesicle populations for dUTP cargo molecules, indicating a more pronounced influence of the additional cleaning process and its associated size-filtering effect, as discussed with reference to Figure 2 and Table 2. This suggests strategies for optimizing sample preparation to increase loading efficiency in drug delivery applications involving size-based filtering of nanovesicle populations around the peak size of the loading yield profile, as further discussed in the following section.

**Figure 4:**
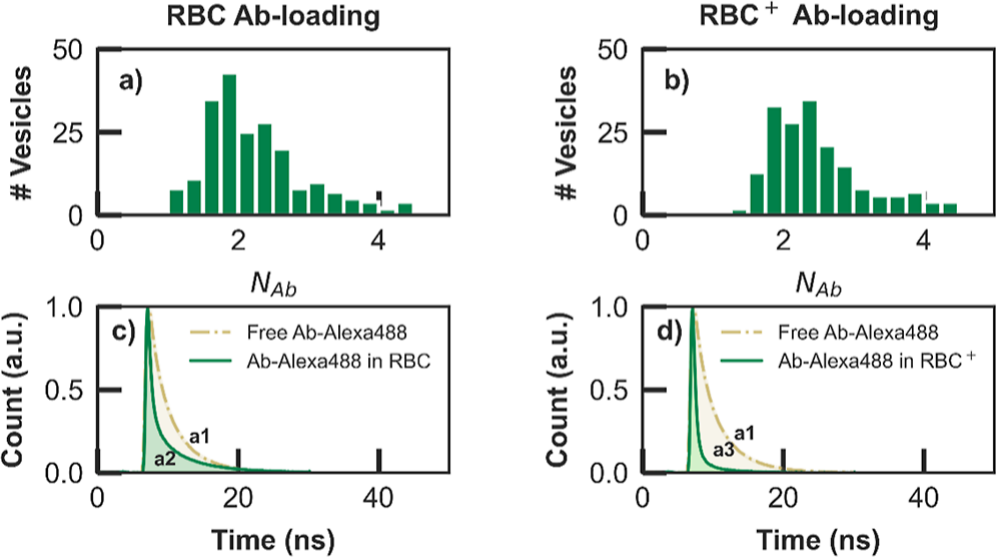
Histograms of a number-normalized brightness per vesicle (*N*_Ab_): (a) RBC and (b) RBC^+^ sample preparations. The normalized lifetime histograms of Alexa488 dye bound to Ab-cargo molecules in three conditions such as a_1_: free in solution (olive dash-dotted), a_2_: loaded into (c) RBC nanovesicles (green solid), and a_3_: loaded into (d) RBC^+^ nanovesicles (green solid).

Finally, the single-molecule resolving capability of fluorescence measurements, combined with further analyses and calibration experiments detailed in S2 and ref (35), afforded also statistical investigations on the number-normalized brightness per vesicle (*N*_Ab_), illustrated in Figure 4a,b. Equivalent data for the dUTP case are presented in S3 (Figure S11), and a comparative summary of the results is provided in Table 2, listing the retrieved values of the maximum number of cargos per loaded nanovesicle (*N*_max_) and its average (*N*_ave_) for all four sample typologies.

The analysis shows that regardless of the type of cargo (Ab or dUTP) and solution cleaning procedure (RBC or RBC^+^), the loaded nanovesicles contain on average two cargo molecules, with excellent agreement between the values obtained for Ab and dUTP in RBC^+^ samples, namely: *N*_max_ = 2.2 and *N*_ave_ = 2.5, confirming the consistency and reliability of the antibody loading process into red-blood-cell-derived nanovesicles.

**Figure 5:**
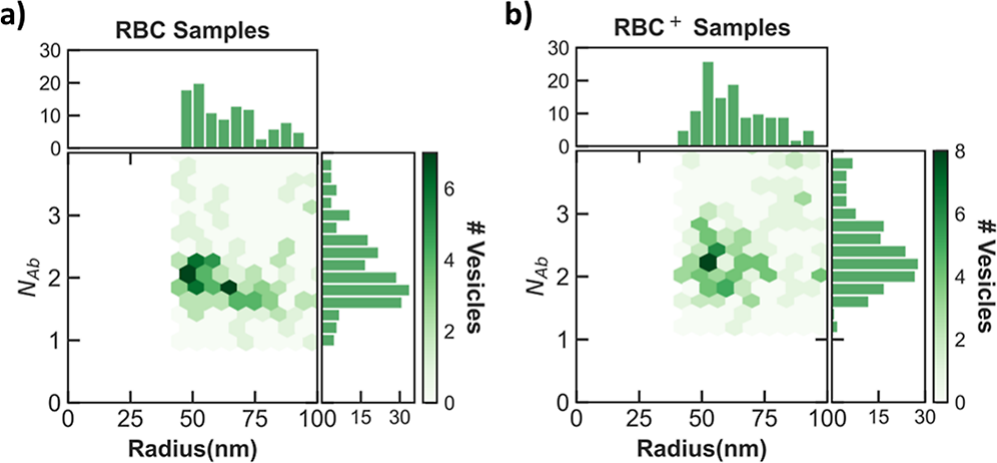
Profile of antibody-loaded nanocarriers: Two-dimensional histograms of loaded nanovesicles versus their radius (*R*) and number-normalized brightness per vesicle (*N*_Ab_), obtained from DC–CFB assessments. The size and number of Ab-cargo molecules per vesicle for the loaded vesicles are depicted in the horizontal and vertical subplots, respectively, for (a) RBC and (b) RBC^+^ preparations. It demonstrates that loaded nanovesicles are mostly populating at a radius of 52 nm and maximum brightness-normalized number of 1.75 and 2.25 Abs per RBC and RBC^+^ nanovesicles, respectively.

Moreover, the calibration measurements required for the retrieval of the statistics of cargo molecules per nanovesicle, as described in S2, revealed unexpected features in the fluorescence signals of Alexa488 dye bound to cargo molecules entrapped into the nanovesicles. Figure 4c,d (Figure S11c,d) illustrates these findings, displaying lifetime histograms of green fluorescence signals from Alexa488 dye bound to Ab (dUTP) molecules in three conditions: (1) free in solution shown by the olive dashed-dotted line, (2) loaded into RBC nanovesicles, and (3) loaded into RBC^+^ nanovesicles, both shown in green solid lines in Figure 4c,d, respectively. The results clearly show a reduction in fluorescence lifetimes for Alexa488 when it is encapsulated in the nanovesicles, whether bound to Ab or dUTP. The observed shortening of lifetimes in Figure 4c,d, which is considered in the results derivation, is a noteworthy effect not highlighted in previous studies so far. Unlike free molecules with monoexponentially decay, τ_lifetime_ = 4 ns for Alexa488 with dUTP and 3.6 ns with Ab, entrapped molecules exhibit a continuous range of shorter lifetimes possibly due to potential Förster resonance energy transfer (FRET) interactions with hemoglobin inside RBC nanovesicles. An alternative explanation could be the occurrence of FRET between Alexa488 dyes and Claret CellVue (far-red dye) employed for labeling the outer membrane of the nanovesicles. Due to the lipophilic nature of this far-red dye, there is a possibility of its partial permeation through the RBC membrane, leading to potential energy interactions with the green dye. Moreover, the additional centrifugation steps during exosome spin column cleaning further exacerbate these FRET effects, resulting in the lowest lifetimes of fluorescent tags particularly in the RBC^+^ case. However, the number and interaction distance of hemoglobin molecules are unpredictable and uncontrollable, leading to varying lifetimes. To account for this effect on the fluorescence brightness of the tagging dye inside RBC and RBC^+^ vesicles (see also S2), the area under their normalized lifetime histograms (a_2_ and a_3_ in Figure 4c,d) is compared with that of free cargo molecules (a_1_ in Figure 4c,d), allowing for the calibration of the average photon count for the entrapped cargos (see Table S3). Conclusively, the DC–CFB assessments provide the two-dimensional profile of loaded vesicles versus radius and Ab-numbers per loaded vesicle, as illustrated in Figure 5, revealing the most populated sizes and the load extent for the Ab-loaded nanovesicles. The load extent represents average values, with a peak at 2.2 and 2.5 for RBC and RBC^+^ preparations, respectively, which suggests that they contained an average of at least two loaded antibody molecules.

Finally, it is worth noting that the additional cleaning step through ESC maintained the loading yields and the average number of antibody cargoes per loaded nanovesicle unchanged. Meanwhile, the evaluations indicated a significant decrease in the average background rates within antibody-loaded vesicles in both the red and green channels. Specifically, these rates decreased approximately 4.6 times in the red channel and 2.0 times in the green channel in RBC^+^ vesicles compared to RBC samples (see Figure S5). These findings underscore the effectiveness of the supplementary purification step in removing more free and nonencapsulated cargoes from the final solution. Particularly in the green channel, this kind of purification results in a reduction of nonencapsulated antibody therapeutics, which can play an essential role in minimizing side effects associated with pharmaceutical drug delivery products.

## 4 Conclusions

In summary, we have demonstrated successful antibody-loading into synthesized nanovesicles from red-blood-cell membranes. Dual-color fluorescence burst investigations at the single-vesicle level revealed a preference for nanovesicles with an average radius of around 65 nm when loaded with Ab-cargos compared to 55 nm obtained in reference experiments performed on dUTP cargos, which aligns with the larger size and molecular weight of Ab molecules. Loading yields for Ab cargos were comparable to those of dUTP-loaded vesicles, peaking at approximately 40%. The optimal vesicle radius of 52 nm and an average loading efficiency of 14% were obtained for Ab-loaded nanovesicles. Considering unexpected lifetime shortening of the Alexa488 fluorophore in produced nanovesicles, likely due to FRET interactions with hemoglobin molecules, revealed an average loading of 2.25 antibody molecules per nanovesicle, consistent with dUTP cargo results under identical conditions. These findings show the loading of nanovesicles with larger antibody therapeutics, showcasing for the first time, to the best of our knowledge, the feasibility of producing antibody-loaded RBC-membrane-derived nanovesicles within a size range similar to that of physiological extracellular vesicles. This holds promise for the development of more biocompatible and scalable intracellular drug delivery nanocarriers with a reduced immunogenicity. These results align well with previous studies utilizing Nanoflow cytometry^45,46^ and single molecule localization microscopy,^47^ which have also observed a similar range of cargo loading in engineered EV subpopulations, with a similar percentage of cargo-loaded extracellular vesicles at the single-molecule and single-vesicle level. Furthermore, the additional cleaning procedures in this study provided stable results for Ab-loading and insights into the relationship between nanovesicle physical properties and cargo nature, offering opportunities to enhance the purity of production yields and customize these nanovesicles for specific therapeutic agent for Ab delivery with substantially lower side effects.

## Supporting Information Available

Details on the sample preparation steps, experimental methods, data analysis procedure, and summary of additional reference results on dUTP-loaded control nanovesicles (PDF)

## Abbreviations

Ab: antibody
AFM: atomic force microscopy
CFB: coincident fluorescence burst
DCFM: dual-color fluorescence microscopy
DRM: detergent resistant membranes
RBC: red blood cell

## References

[ref1] Carter P. J.; Rajpal A. Designing antibodies as therapeutics. Cell 2022, 185 (15), 2789–2805. 10.1016/j.cell.2022.05.029. 35868279

[ref2] Trkulja C. L.; Jungholm O.; Davidson M.; Jardemark K.; Marcus M. M.; Hägglund J.; Karlsson A.; Karlsson R.; Bruton J.; Ivarsson N.; Srinivasa S. P.; Cavallin A.; Svensson P.; Jeffries G. D. M.; Christakopoulou M.-N.; Reymer A.; Ashok A.; Willman G.; Papadia D.; Johnsson E.; Orwar O. Rational antibody design for undruggable targets using kinetically controlled biomolecular probes. Sci. Adv. 2021, 7 (16), eabe6397 10.1126/sciadv.abe6397. 33863724 PMC8051879

[ref3] Mimura Y.; Saldova R.; Mimura-Kimura Y.; Rudd P. M.; Jefferis R. Importance and Monitoring of Therapeutic Immunoglobulin G Glycosylation. Exper. Suppl. 2021, 112, 481–517. 10.1007/978-3-030-76912-3_15. 34687020

[ref4] Li S.; McCraw A. J.; Gardner R. A.; Spencer D. I. R.; Karagiannis S. N.; Wagner G. K. Glycoengineering of Therapeutic Antibodies with Small Molecule Inhibitors. Antibodies 2021, 10 (4), 44 10.3390/antib10040044. 34842612 PMC8628514

[ref5] Yuan P.; Yang F.; Liew S. S.; Yan J.; Dong X.; Wang J.; Du S.; Mao X.; Gao L.; Yao S. Q. Intracellular Co-delivery of native antibody and siRNA for combination therapy by using biodegradable silica nanocapsules. Biomaterials 2022, 281, 121376 10.1016/j.biomaterials.2022.121376. 35065331

[ref6] Zhang G.; Zhang J.; Gao Y.; Li Y.; Li Y. Strategies for targeting undruggable targets. Expert Opin. Drug Discovery 2022, 17 (1), 55–69. 10.1080/17460441.2021.1969359. 34455870

[ref7] Lyu X.; Zhao Q.; Hui J.; Wang T.; Lin M.; Wang K.; Zhang J.; Shentu J.; Dalby P. A.; Zhang H.; Liu B. The global landscape of approved antibody therapies. Antibiot. Ther. 2022, 5 (4), 233–257. 10.1093/abt/tbac021. PMC9535261 36213257

[ref8] Trenevska I.; Li D.; Banham A. H. Therapeutic Antibodies against Intracellular Tumor Antigens. Front. Immunol. 2017, 8, 1001 10.3389/fimmu.2017.01001. 28868054 PMC5563323

[ref9] Lagassé H. D.; Alexaki A.; Simhadri V. L.; Katagiri N. H.; Jankowski W.; Sauna Z. E.; Kimchi-Sarfaty C. Recent advances in (therapeutic protein) drug development. F1000Research 2017, 6, 113 10.12688/f1000research.9970.1. 28232867 PMC5302153

[ref10] Koch K. C.; Tew G. N. Functional Antibody Delivery: Advances in Cellular Manipulation. Adv. Drug Delivery Rev. 2023, 192, 114586 10.1016/j.addr.2022.114586. 36280179

[ref11] Niamsuphap S.; Fercher C.; Kumble S.; Huda P.; Mahler S. M.; Howard C. B. Targeting the undruggable: emerging technologies in antibody delivery against intracellular targets. Expert Opin. Drug Delivery 2020, 17 (9), 1189–1211. 10.1080/17425247.2020.1781088. 32524851

[ref12] Hershman R. L.; Li Y.; Ma F.; Xu Q.; Van Deventer J. A. Intracellular Delivery of Antibodies for Selective Cell Signaling Interference. ChemMedChem 2022, 17 (6), e202100678 10.1002/cmdc.202100678. 34890114

[ref13] Gaston J.; Maestrali N.; Lalle G.; Gagnaire M.; Masiero A.; Dumas B.; Dabdoubi T.; Radošević K.; Berne P.-F. Intracellular delivery of therapeutic antibodies into specific cells using antibody-peptide fusions. Sci. Rep. 2019, 9 (1), 18688 10.1038/s41598-019-55091-0. 31822703 PMC6904672

[ref14] Singh K.; Ejaz W.; Dutta K.; Thayumanavan S. Antibody Delivery for Intracellular Expert Opin. Drug DeliveryTargets: Emergent Therapeutic Potential. Bioconjugate Chem. 2019, 30 (4), 1028–1041. 10.1021/acs.bioconjchem.9b00025. PMC6470022 30830750

[ref15] Zhang Y.; Yu L. C. Single-cell microinjection technology in cell biology. Bioessays 2008, 30 (6), 606–610. 10.1002/bies.20759. 18478541

[ref16] Tompers D. M.; Labosky P. A. Electroporation of murine embryonic stem cells: a step-by-step guide. Stem Cells 2004, 22 (3), 243–249. 10.1634/stemcells.22-3-243. 15153600

[ref17] Slastnikova T. A.; Ulasov A. V.; Rosenkranz A. A.; Sobolev A. S. Targeted Intracellular Delivery of Antibodies: The State of the Art. Front. Pharmacol 2018, 9, 1208 10.3389/fphar.2018.01208. 30405420 PMC6207587

[ref18] Li Y.; Li P.; Li R.; Xu Q. Intracellular Antibody Delivery Mediated by Lipids, Polymers, and Inorganic Nanomaterials for Therapeutic Applications. Adv. Ther. 2020, 3 (12), 2000178 10.1002/adtp.202000178.

[ref19] Kim A.; Miura Y.; Ishii T.; Mutaf O. F.; Nishiyama N.; Cabral H.; Kataoka K. Intracellular Delivery of Charge-Converted Monoclonal Antibodies by Combinatorial Design of Block/Homo Polyion Complex Micelles. Biomacromolecules 2016, 17 (2), 446–453. 10.1021/acs.biomac.5b01335. 26691492

[ref20] Hirai Y.; Hirose H.; Imanishi M.; Asai T.; Futaki S. Cytosolic protein delivery using pH-responsive, charge-reversible lipid nanoparticles. Sci. Rep. 2021, 11 (1), 19896 10.1038/s41598-021-99180-5. 34615928 PMC8494842

[ref21] Gao L.; Han L.; Ding X.; Xu J.; Wang J.; Zhu J.; Lu W.; Sun J.; Yu L.; Yan Z.; Wang Y. An effective intracellular delivery system of monoclonal antibody for treatment of tumors: erythrocyte membrane-coated self-associated antibody nanoparticles. Nanotechnology 2017, 28 (33), 335101 10.1088/1361-6528/aa7c43. 28657549

[ref22] Zhou H.; Fan Z.; Lemons P. K.; Cheng H. A Facile Approach to Functionalize Cell Membrane-Coated Nanoparticles. Theranostics 2016, 6 (7), 1012–1022. 10.7150/thno.15095. 27217834 PMC4876625

[ref23] Rao L.; Bu L. L.; Xu J. H.; Cai B.; Yu G. T.; Yu X.; He Z.; Huang Q.; Li A.; Guo S. S.; Zhang W. F.; Liu W.; Sun Z. J.; Wang H.; Wang T. H.; Zhao X. Z. Red Blood Cell Membrane as a Biomimetic Nanocoating for Prolonged Circulation Time and Reduced Accelerated Blood Clearance. Small 2015, 11 (46), 6225–6236. 10.1002/smll.201502388. 26488923

[ref24] Li J.-Q.; Zhao R.-X.; Yang F.-M.; Qi X.-T.; Ye P.-K.; Xie M. An erythrocyte membrane-camouflaged biomimetic nanoplatform for enhanced chemo-photothermal therapy of breast cancer. J. Mater. Chem. B 2022, 10 (12), 2047–2056. 10.1039/D1TB02522H. 35254366

[ref25] Wang H.; Liu Y.; He R.; Xu D.; Zang J.; Weeranoppanant N.; Dong H.; Li Y. Cell membrane biomimetic nanoparticles for inflammation and cancer targeting in drug delivery. Biomater. Sci. 2020, 8 (2), 552–568. 10.1039/C9BM01392J. 31769765

[ref26] Caracciolo G. Clinically approved liposomal nanomedicines: lessons learned from the biomolecular corona. Nanoscale 2018, 10 (9), 4167–4172. 10.1039/C7NR07450F. 29450412

[ref27] El Sayed M. M.; Takata H.; Shimizu T.; Kawaguchi Y.; Abu Lila A. S.; Elsadek N. E.; Alaaeldin E.; Ishima Y.; Ando H.; Kamal A.; Sarhan H. A.; Ishida T. Hepatosplenic phagocytic cells indirectly contribute to anti-PEG IgM production in the accelerated blood clearance (ABC) phenomenon against PEGylated liposomes: Appearance of an unexplained mechanism in the ABC phenomenon. J. Controlled Release 2020, 323, 102–109. 10.1016/j.jconrel.2020.04.011. 32278827

[ref28] Mohamed M.; Abu Lila A. S.; Shimizu T.; Alaaeldin E.; Hussein A.; Sarhan H. A.; Szebeni J.; Ishida T. PEGylated liposomes: immunological responses. Sci. Technol. Adv. Mater. 2019, 20 (1), 710–724. 10.1080/14686996.2019.1627174. 31275462 PMC6598536

[ref29] Han X.; Shen S.; Fan Q.; Chen G.; Archibong E.; Dotti G.; Liu Z.; Gu Z.; Wang C. Red blood cell-derived nanoerythrosome for antigen delivery with enhanced cancer immunotherapy. Sci. Adv. 2019, 5 (10), eaaw6870 10.1126/sciadv.aaw6870. 31681841 PMC6810293

[ref30] Malhotra S.; Dumoga S.; Sirohi P.; Singh N. Red Blood Cells-Derived Vesicles for Delivery of Lipophilic Drug Camptothecin. ACS Appl. Mater. Interfaces 2019, 11 (25), 22141–22151. 10.1021/acsami.9b04827. 31148443

[ref31] Burger P.; Hilarius-Stokman P.; de Korte D.; van den Berg T. K.; van Bruggen R. CD47 functions as a molecular switch for erythrocyte phagocytosis. Blood 2012, 119 (23), 5512–5521. 10.1182/blood-2011-10-386805. 22427202

[ref32] Ma H.; Ó’Fágáin C.; O’Kennedy R. Antibody stability: A key to performance - Analysis, influences and improvement. Biochimie 2020, 177, 213–225. 10.1016/j.biochi.2020.08.019. 32891698

[ref33] Dubois L.; Löf L.; Larsson A.; Hultenby K.; Waldenström A.; Kamali-Moghaddam M.; Ronquist G.; Ronquist K. G. Human erythrocyte-derived nanovesicles can readily be loaded with doxorubicin and act as anticancer agents. Cancer Res. Front. 2018, 4, 13–26. 10.17980/2018.13.

[ref34] Malhotra S.; Dumoga S.; Singh N. Red blood cells membrane-derived nanoparticles: Applications and key challenges in their clinical translation. Wiley Interdiscip. Rev.: Nanomed. Nanobiotechnol. 2022, 14 (3), e1776 10.1002/wnan.1776. 35106966

[ref35] Sanaee M.; Sandberg E.; Ronquist K. G.; Morrell J. M.; Widengren J.; Gallo K. Coincident Fluorescence-Burst Analysis of the Loading Yields of Exosome-Mimetic Nanovesicles with Fluorescently-Labeled Cargo Molecules. Small 2022, 18 (12), 2106241 10.1002/smll.202106241. 35084110

[ref36] Garg A.; Malhotra R.; Urs A. B. Ghost cells unveiled: A comprehensive review. J. Oral Biosci. 2022, 64 (2), 202–209. 10.1016/j.job.2022.03.005. 35398253

[ref37] Hoffman J. F. On red blood cells, hemolysis and resealed ghosts. Adv. Exp. Med. Biol. 1992, 326, 1–15. 10.1007/978-1-4615-3030-5_1. 1295293

[ref38] Zade-Oppen A. M. M. Posthypertonic Hemolysis in Sodium Chloride Systems. Acta Physiol. Scand. 1968, 73 (3), 341–364. 10.1111/j.1365-201x.1968.tb10873.x. 5709591

[ref39] Welton J. L.; Webber J. P.; Botos L. A.; Jones M.; Clayton A. Ready-made chromatography columns for extracellular vesicle isolation from plasma. J. Extracell. Vesicles 2015, 4, 27269 10.3402/jev.v4.27269. 25819214 PMC4376847

[ref40] Schwille P.; Meyer-Almes F. J.; Rigler R. Dual-color fluorescence cross-correlation spectroscopy for multicomponent diffusional analysis in solution. Biophys. J. 1997, 72 (4), 1878–1886. 10.1016/S0006-3495(97)78833-7. 9083691 PMC1184381

[ref41] Feng Y.; Liu M.; Li X.; Li M.; Xing X.; Liu L. Nanomechanical Signatures of Extracellular Vesicles from Hematologic Cancer Patients Unraveled by Atomic Force Microscopy for Liquid Biopsy. Nano Lett. 2023, 23 (4), 1591–1599. 10.1021/acs.nanolett.3c00093. 36723485

[ref42] Ridolfi A.; Brucale M.; Montis C.; Caselli L.; Paolini L.; Borup A.; Boysen A. T.; Loria F.; van Herwijnen M. J. C.; Kleinjan M.; Nejsum P.; Zarovni N.; Wauben M. H. M.; Berti D.; Bergese P.; Valle F. AFM-Based High-Throughput Nanomechanical Screening of Single Extracellular Vesicles. Anal. Chem. 2020, 92 (15), 10274–10282. 10.1021/acs.analchem.9b05716. 32631050

[ref43] Jena B. P.; Stemmer P. M.; Wang S.; Mao G.; Lewis K. T.; Walz D. A. Human Platelet Vesicles Exhibit Distinct Size and Proteome. J. Proteome Res. 2017, 16 (7), 2333–2338. 10.1021/acs.jproteome.7b00309. 28587468 PMC6844074

[ref44] Yakubovich E. I.; Polischouk A. G.; Evtushenko V. I. Principles and Problems of Exosome Isolation from Biological Fluids. Biochem. (Moscow), Suppl. Ser. 2022, 16 (2), 115–126. 10.1134/S1990747822030096. PMC9202659 35730027

[ref45] Silva A. M.; Lázaro-Ibáñez E.; Gunnarsson A.; Dhande A.; Daaboul G.; Peacock B.; Osteikoetxea X.; Salmond N.; Friis K. P.; Shatnyeva O.; Dekker N. Quantification of protein cargo loading into engineered extracellular vesicles at single-vesicle and single-molecule resolution. J. Extracell. Vesicles 2021, 10 (10), e12130 10.1002/jev2.12130. 34377376 PMC8329990

[ref46] Chen C.; Sun M.; Wang J.; Su L.; Lin J.; Yan X. Active cargo loading into extracellular vesicles: Highlights the heterogeneous encapsulation behaviour. J. Extracell. Vesicles 2021, 10 (13), e12163 10.1002/jev2.12163. 34719860 PMC8558234

[ref47] Puthukodan S.; Hofmann M.; Mairhofer M.; Janout H.; Schurr J.; Hauser F.; Naderer C.; Preiner J.; Winkler S.; Sivun D.; Jacak J. Purification Analysis, Intracellular Tracking, and Colocalization of Extracellular Vesicles Using Atomic Force and 3D Single-Molecule Localization Microscopy. Anal. Chem. 2023, 95 (14), 6061–6070. 10.1021/acs.analchem.3c00144. 37002540 PMC10100414

