# Supplementary material for: Antibody-Loading of Biological Nanocarrier Vesicles Derived from Red-Blood-Cell Membranes
Source: ACS Omega. 2024 May 14;9(21):22711–8. doi: 10.1021/acsomega.4c00650 (PMC11137724; doi:10.1021/acsomega.4c00650)
Supplement: Supplementary file 1 — ao4c00650_si_001.pdf [file ao4c00650_si_001.pdf]

Supporting Information

Antibody-loading of biological nanocarrier vesicles derived from red-blood-cell membranes

Maryam Sanaee,[1]* *K. Göran Ronquist,*[2] *Elin Sandberg,* [1] *Jane M. Morrell,*[2] *Jerker Widengren,* [1] *and Katia Gallo*[1]

[1] Department of Applied Physics, School of Engineering Sciences, KTH Royal Institute of Technology, Stockholm 10691, Sweden.

[2] Department of Clinical Sciences, Swedish University of Agricultural Sciences, Uppsala 75007, Sweden

*Corresponding author. Email: msanaee@kth.se

Contents

S1- Experimental methods........S3

S1-1- Sample preparation........S3

S1-2- Atomic force microscopy measurements........S6

S1-3- Dual-color fluorescence microscopy experiments........S6

S2- Data analysis........S7

S2-1- Background calculation........S8

S2-2- Assessment of total population of nanovesicles........S9

2-3- Loaded nanovesicles........S10

2-4- The lifetimes and brightness assessments........S13

S3- Reference dUTP results and comparative summary with Ab-loading........S13

Reference........S17

S1- Experimental methods

A summary of the experimental methods employed during this research are provided in following subsections including the preparation description of the sample preparation, the AFM and dual-color fluorescence microscopy measurements.

S1-1- Sample preparation

The preparation of Ab-loaded red blood cell (RBC) nanovesicles includes five main steps and is briefly described below. They follow closely the previously developed procedures with dUTP-cargo molecules [1].

a) Red Blood Cell ghosts' preparation

Following the acquisition of blood bags containing red blood cells from Uppsala University Hospital, a 10 mL volume of red blood cells was washed 3 times (1:5 ratio) in phosphate buffered saline (PBS), by centrifugation (Nino lab Heraeus) at 2100g and 4°C for 10 min. Washed red blood cells were lysed in hypotonic phosphate buffer (53.4 mOsmol.L^{-1}). Most of the hemoglobin was removed by cyclic steps of centrifugation/dilution at 20,000g and 4°C for 30min, Beckman Coulter (BC), SW32Ti rotor) Derived RBC ghosts were stored at -20°C and utilized within a timeframe of less than three months.

b) DRM vesicles isolation

Stored RBC ghosts (10 mL) were pelleted by ultracentrifugation at 100,000g and 4°C for 1h, (BC, SW32Ti). The pellet was dissolved in PBS containing 1% triton x-100 and incubated on ice for 30 min before separation on density gradient built by 40%, 30%, 24%, and 10% sucrose, run at 230,000g and 4°C for 5h, (BC, SW40Ti rotor). Fractions on top of 24% and 30% sucrose (density of 1.10 and 1.13 mg.cm^{-3}) were collected and in later separations the 24% density was skipped. Extracted fractions were pelleted in PBS containing 10% sucrose at 150,000g and 4°C for 1h, (BC, SW32Ti). Pellets, sucrose charged detergent resistant membrane nanovesicles, DRM-nanovesicles, were stored at -20°C.

c) DRM-nanovesicle loading with Alexa 488 antibody

The central procedures involved in loading Ab cargo into DRM-nanovesicles, following the final preparation steps are illustrated in Figure S1. The frozen DRM-nanovesicle pellet was dissolved in 10µL goat anti-Chicken IgY (H+L) secondary antibody tagged with Alexa488 (Ab-Alexa488 Thermo Fisher), and after a short incubation diluted in PBS. The shift from hypertonicity caused by encapsulated sucrose to isotonicity due to Ab : PBS dilution led to "post-hypertonic lysis" [2], triggering the rupture of nanovesicles, followed by re-vesiculation in the Ab enriched PBS solution, all fluorescently labeled samples were shielded from direct light exposure. Ab-loaded DRM-nanovesicles were pelleted in low

sucrose (1-3%) by ultracentrifugation at 100,000g and 4°C for 1h (BC, SW32Ti) to remove excess fluorescence.

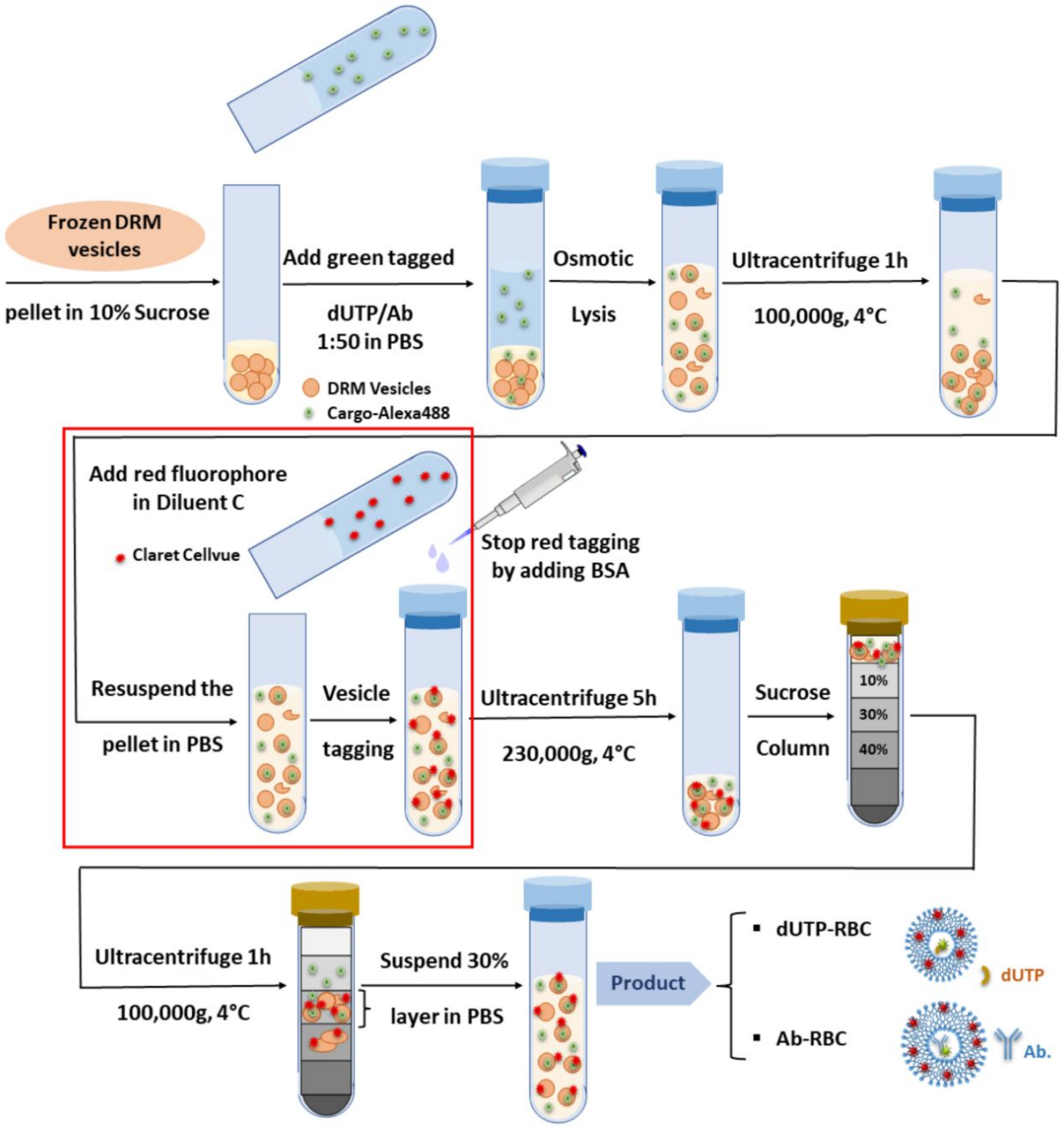

Figure S1. The schematic of the core processes associated with the loading of Ab-Alexa488 cargos into DRM nanovesicles derived from RBC-membrane, including the final preparation stages such as red fluorescence tagging of vesicles, highlighted in red box.

d) Red fluorescence staining of DRM-nanovesicles

In Figure S1, the process for staining loaded nanovesicles with red fluorescence is highlighted within a red box. To label the vesicle membranes, the pellets were mixed with CellVue Claret membrane kit-staining-component Diluent C (Sigma-Aldrich, Miniclaret-1kt), following the manufacturer's instructions. The staining reaction was stopped by 3% bovine serum albumin in PBS and immediately

pelleted by top-loading on a low concentrated sucrose solution (1-3%) at 100,000g and 4°C for 1h (BC, SW32Ti). The pellets of DRM-nanovesicles preparation were resuspended in PBS and kept at 4°C in dark for physics experiments to be conducted. Short summary, this approach is applied to investigate the efficiency of Ab loading of the prepared samples and compare them with dUTP cargo. To reach this goal, the cargo molecules were fluorescently stained by the dye Alexa488 with excitation and detection wavelengths at 485 nm and 535 nm, respectively, while nanovesicle membranes were tagged by the far-red dye, CellVue Claret, with corresponding excitation and detection wavelengths of 640 nm and 720 nm, respectively.

e) Extra cleaning with exosome spin column

In the last phase of the preparation, to evaluate the impact of additional purification on the loading efficiency and cleanliness of loaded RBC nanovesicles, a portion of these loaded DRM-nanovesicles underwent a purification process utilizing the exosome spin column (ESC, Thermo Fisher, ESC MW 3000).

In Figure S2, the real pictures of the prepared samples in different steps are illustrated, including a) the top-loading of purified erythrocyte ghosts in 1% triton x-100 on density gradient and several formed sucrose buoyancies (10%, 24%, 30% and 40%), b) the separated erythrocyte ghosts on density gradient after being treated with 1% triton x-100, while the upper small band is detergent resistant membranes and the broader lower band is bulk erythrocyte membrane which are not floating up as the lighter DRM-fraction, c) DRM-nanovesicles loaded with Ab-Alex488, and finally d) the DRM-nanovesicles stained with far red namely as Ab-RBC.

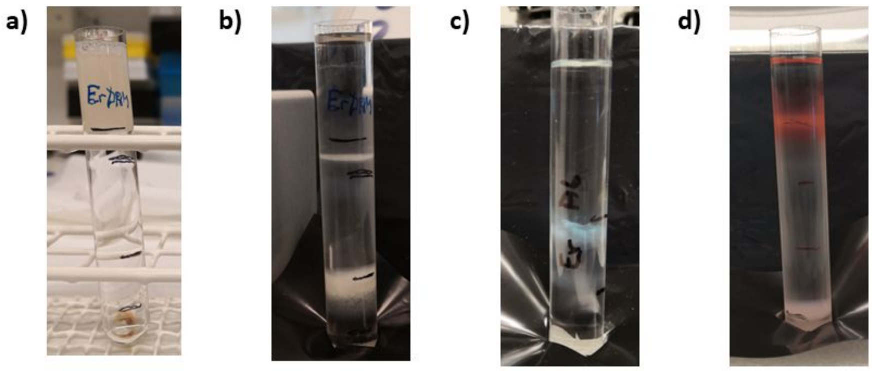

Figure S2. The real picture of the samples during each preparation steps encompassing a) the top-loading of purified erythrocyte ghosts in 1% triton x-100 on density gradient, b) the separated erythrocyte ghosts on density gradient after being treated with 1% triton x-100, while the upper small band is detergent resistant membranes and the broader lower band is bulk erythrocyte membrane that are not floating up as the lighter DRM-fraction, c)

DRM-nanovesicles loaded with Ab-Alex488, and d) the DRM-nanovesicles stained with far red as final product (Ab-RBC).

S1-2- Atomic force microscopy measurements

The morphology of individual vesicles and their size distribution were assessed using a commercial atomic force microscope (FastScan Bruker AFM). The AFM operated at a slow scan rate of 0.5 Hz and was set to tapping mode under ambient air conditions. NCHV-A (Bruker) cantilevers with a tip featuring an average radius of 8 nm were employed for these measurements. Sample handling and AFM measurements for Ab-loaded samples followed a methodology similar to that recently reported for dUTP-loaded nanovesicles [1]. The AFM-derived size histograms were compared to size histograms of the complete vesicle populations determined through single-color red fluorescence analysis which is explained in next section.

S1-3- Dual-color fluorescence microscopy experiments

The dual-color fluorescence microscopy (DCFM) experiments were conducted using a custom-built setup based on a commercial confocal microscope (Olympus FV1200) equipped with a water immersion objective (60x, NA 1.2, Olympus, UPlanSApo) [1]. Figure S2 depicts the main components of this microscope. Two lasers operating at wavelengths of 485 nm (Picoquant LDH-D-C-485) and 640 nm (LDH-D-C-640) were Picosecond-pulsed modulated at 20 MHz repetition rate using pulsed-interleaved mode for sample excitation to avoid dual-color cross talk (Fig. S2 b). The lasers power was adjusted to maintain a consistent excitation of 80 μW at the back-focal plane of the objective during all measurements. In the detection pathway, the signals emitted by the red and green fluorophores were passed through a 50 μm pinhole and spatially separated by a dichroic mirror. The emitted red photons were further filtered through an HQ720/150 (Chroma) filter, and the green photons passed through an HQ535/70 (Chroma) filter. The fluorescent light emitted at each wavelength was directed onto either Picoquant tau-spad or Perkin & Elmer (SPCM-AQR-14) single photon-counting avalanche detectors (SPADs), connected to a time-correlated single-photon counting (TCSPC) module (HydraHarp 400) equipped with suitable software (Symphotime, Picoquant) for data acquisition and analysis. Prior to each experimental session, calibration measurements were conducted using well-characterized fluorophores like Cy5 and Rhodamine110 as references to confirm the consistency of the measurement settings and to determine the focal detection volume at both red and green excitation wavelengths, which were found to be 0.320 and 0.165 femtoliters, respectively. A volume of 100 μL (approximately 2 μg) of the nanovesicle solution (diluted 1:100 in PBS) was applied to an eight-well container on top of the microscope objective. These measurements were carried out for a time duration longer than 50 min at a constant temperature in a completely dark room [1].

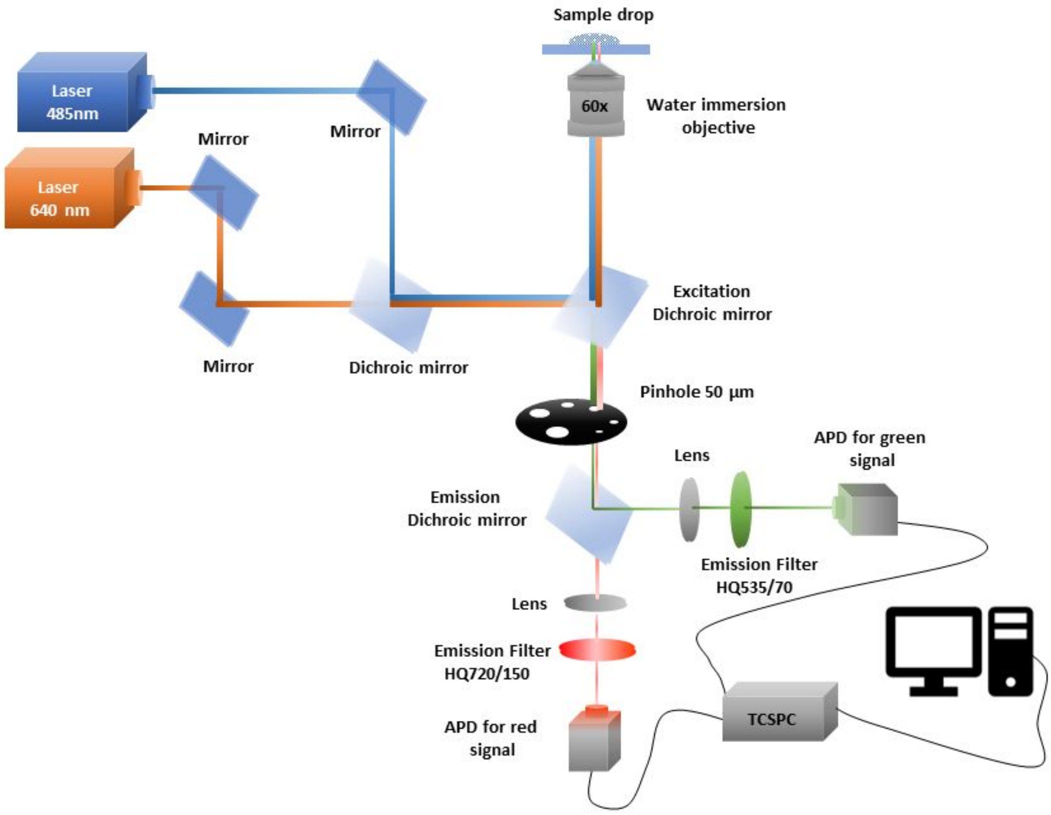

Figure S3. The essential elements of dual-color fluorescence microscope include two lasers emitting at wavelengths of 485 nm and 640 nm utilizing, the Picoquant LDH-D-C-485 and LDH-D-C-640 lasers, respectively. Two lasers were synchronized and operated in a picosecond pulse (shown with sharp red and blue gaussian pulses) modulated at repetition frequency of 20 MHz, utilizing pulsed-interleaved mode, to excite the red and green fluorophores separately in time, while the collected fluorescence signals were time gated through highlighted red and green time windows.

S2- Data analysis

The dual-color coincident fluorescence burst (DC-CFB) analysis was developed recently [1] and is applied here to rigorously assess the loading efficiency of Ab-RBC and Ab-RBC^+ preparations. To that aim, the collected red and green fluorescence signals were time-gated according to their excitation pulses and fluorescence lifetimes, as shown in Figure S4a-d, where the red and green regions highlight the time windows for photon collection in the relevant spectral ranges.

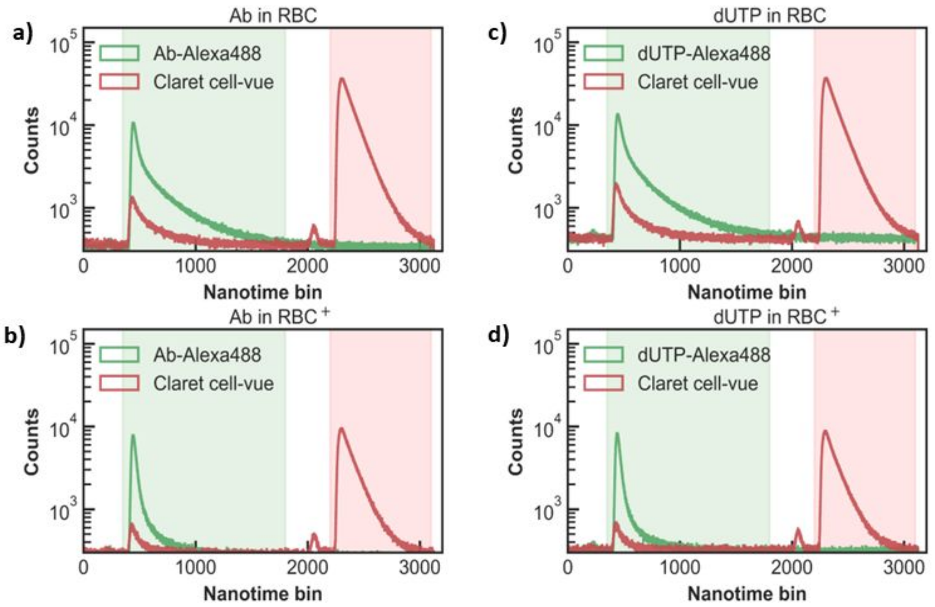

Figure S4. The time resolved lifetime histograms of fluorescent green and red channels highlighting their respective lifetimes (Nanotime bin=16 ps) and time gating windows (shaded green and red areas) for: a-b) Ab-loaded and c- d) dUTP-loaded RBC and RBC^+ preparations.

S2-1- Background calculation

Before searching for the fluorescent bursts at each color, the background rates versus time were evaluated for each preparation at 50 s temporal intervals, as depicted in Figure S5.

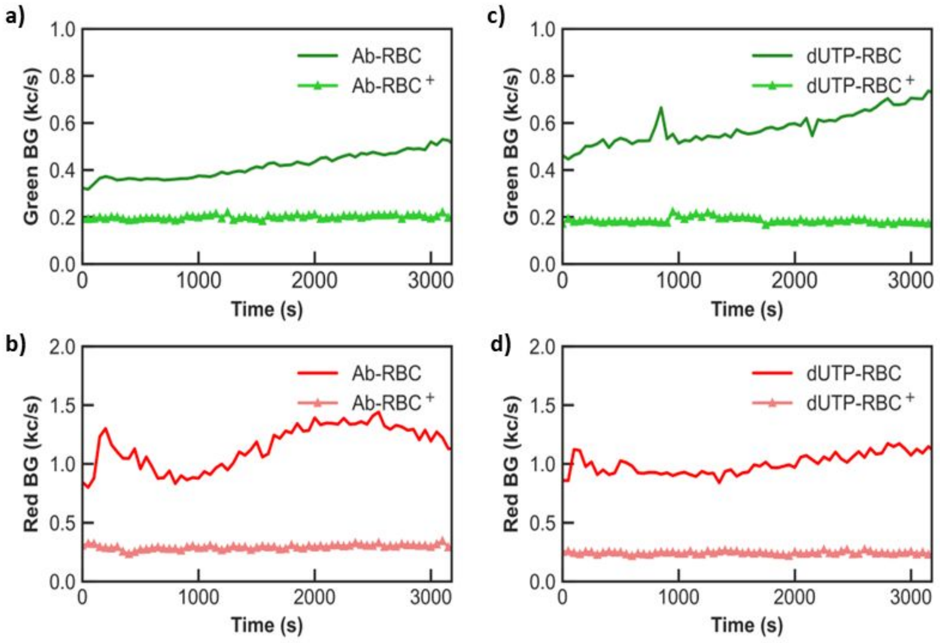

Figure S5. Calibration measurements of the background rates (BG) for the green and red channels performed in four different experimental sessions, on: a-b) Ab- and c-d) dUTP- loaded nanovesicles for samples without (RBC, solid lines) and with (RBC^{+}, line with markers) additional cleaning steps (see S1).

In summary, the evaluations reveal that the average background rates in antibody (dUTP)-loaded vesicles decreased approximately 4.6 (4.4) times in the red channel and 1.95 (2.75) times in the green channel after the additional cleaning process. In conclusion, these results underscore the efficacy of the supplementary cleaning step in eliminating more non-encapsulated cargoes from the final solution. Remarkably, this cleaning process had a substantial impact on the green channel, leading to a fourfold reduction in its corresponding background rates. This reduction is primarily attributed to the removal of non-encapsulated antibody/dUTP cargoes.

S2-2- Assessment of total population of nanovesicles

Following the subtraction of time-dependent background rates from the red and green channels, the DC-CFB analytical method was employed to identify the red and green fluorescence bursts, corresponding to the entire nanovesicle population and the green-tagged cargo molecules, whether located inside or outside the RBC nanovesicles, respectively. Based on the Stokes–Einstein diffusion theory, each burst duration time (τ_{burst}) in the fluorescence time traces obtained from the experiments was converted into the radius (R) of the nanovesicles or molecules on single-vesicle and single-molecule basis. This conversion was accomplished as $R = \frac{4k_B T}{6\pi\mu w_0^2}\tau_{burst}$, where k_B, T and μ represent the Boltzmann constant, the lab temperature, and the viscosity of the solvent (water), respectively. The lateral radius

of the confocal volume, denoted as w_0, was measured in calibration experiments as $w_{0r} = 319\ nm$ and $w_{0g} = 254\ nm$ for the red and green laser spots, respectively.

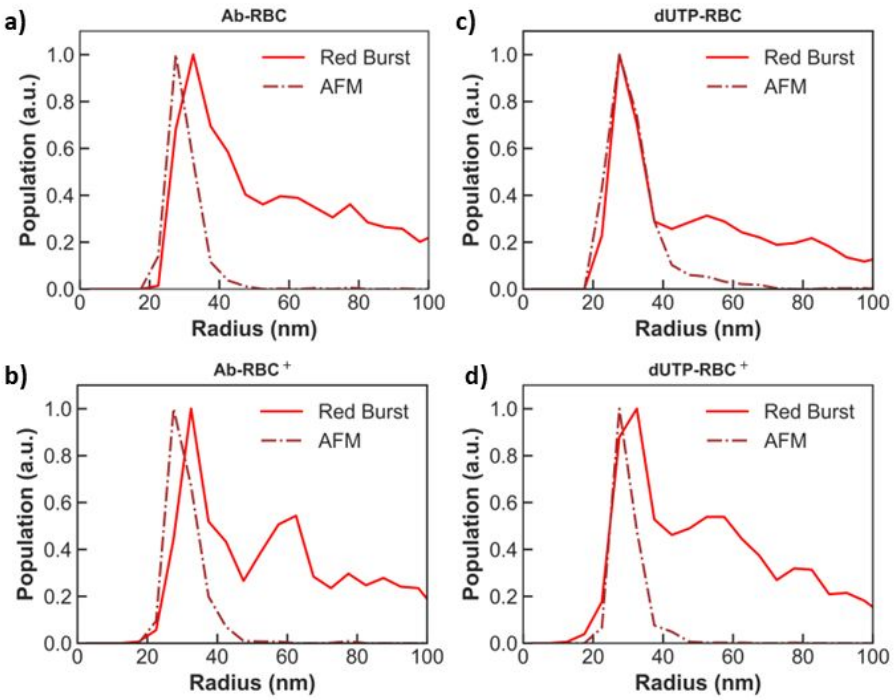

Figure S6. Nanovesicle size distributions normalized to their peak value obtained by AFM (dash-dotted brown) and fluorescence red burst analyses (solid red) for: Ab-loaded a) RBC, b) RBC^+, dUTP-loaded c) RBC and d) RBC^+ preparations.

Therefore, the determination of the total nanovesicle population involved identifying optimized key parameters for the DC-CFB analysis, including minimum photon number (M) and count rate (F) thresholds for both red and green bursts. As previously detailed, the optimal settings for M_r and F_r for the red bursts were established through a burst analysis protocol that systematically compared the red fluorescence results with independent AFM measurements to validate the extracted size distributions. Meanwhile, there might be some differences between AFM and red fluorescence size distributions due to hydration effect in the latter one as it is conducted in physiological buffer solution. The outcomes of the total population assessment using the red fluorescence signal (N_{tot}) and their comparison with corresponding AFM size profiles are depicted in Figure S6, for two different preparations (RBC and RBC^+) and cargo molecules (Ab and dUTP).

2-3- Loaded nanovesicles

The optimized burst conditions for green channel (M_g and F_g) were found based on the established protocol in DC-CFB analysis, by searching for coincident-red and coincident-green bursts in

synchronized dual-color fluorescence time traces. According to this methodology, the coincident-red and coincident-green size distributions versus vesicles' radius (R) were optimized to get minimum difference ($\boldsymbol{\varepsilon}(\boldsymbol{R})$) between their normalized distributions as their results are depicted in Figure S7 and S7, for both Ab- and dUTP-loaded preparations, respectively. Subsequently, the size distribution of coincident red is attributed to the sub-population of loaded nanovesicles (N_{load}). The summary of the optimized burst conditions for red and green channels is provided in Table S1.

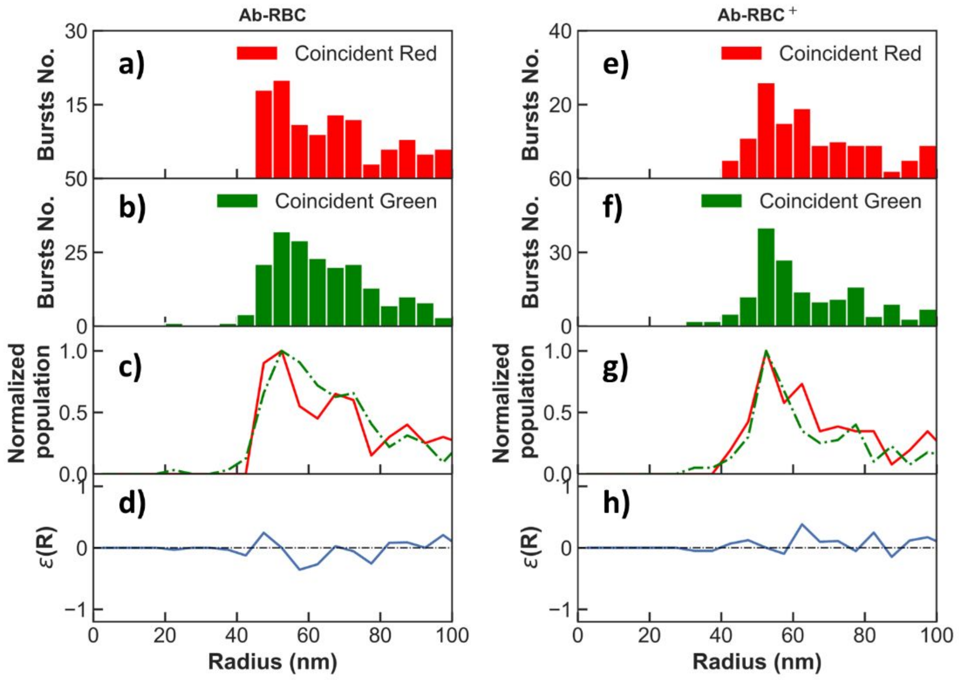

Figure S7. Loaded nanovesicle size histograms retrieved from the fluorescence experiments for a-c) Ab-RBC and e-g) Ab-RBC^{+} preparations. a, e) coincident-red and b, f) coincident-green analyses, with the procedures and methodologies defined in Ref. 1 and corresponding c, g) normalized histograms of the nanovesicle distributions, where coincident-, red and green results are highlighted by solid red and dash-dotted green lines, respectively. d, h) difference between the latter curves in the case of c, g), respectively, providing the error ($\boldsymbol{\varepsilon}(\boldsymbol{R})$) minimization used for optimizing the burst data, as explained in Ref. 1.

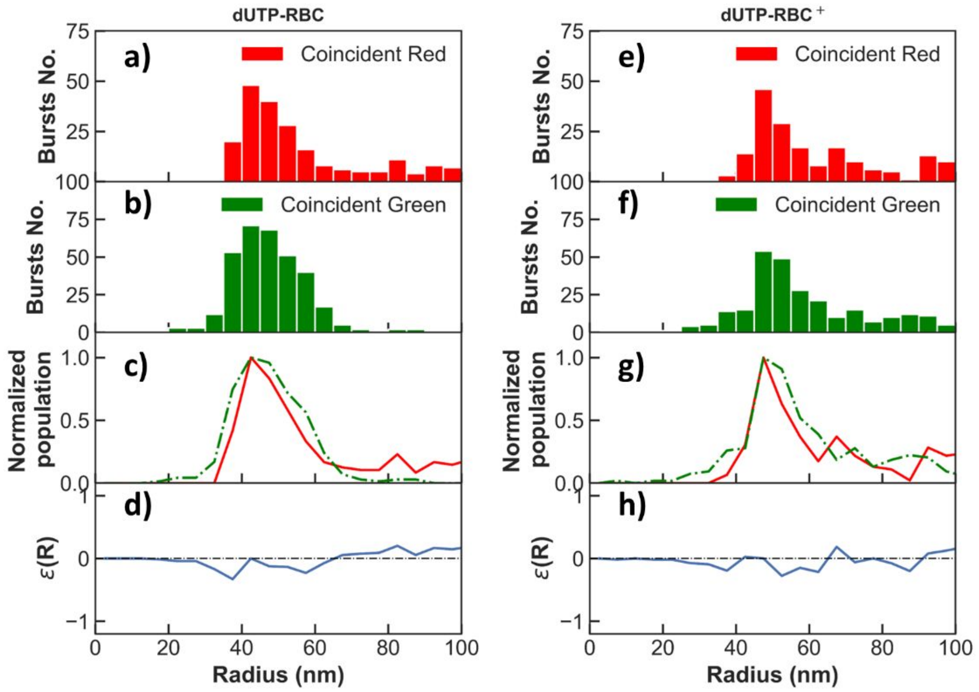

Figure S8. Size histograms of loaded nanovesicles obtained from fluorescence experiments for a-c) dUTP-RBC and e-g) dUTP-RBC$^+$ preparations. In a, e), the histograms are derived from red fluorescence analyses, while in b, f), their result from coincident green analyses are provided, following the procedures and methodologies outlined in Ref. 1. Corresponding c, g) depict normalized histograms of the nanovesicle distributions, derived through coincident-red and coincident-green results, highlighted by solid red and dash-dotted green lines, respectively. In d, h), the difference between the curves presented at c, g), respectively, serving as the basis for error minimization utilized in the optimization of burst data, as detailed in Ref. 1.

Sample	Ab-RBC		Ab-RBC$^+$		dUTP-RBC		dUTP-RBC$^+$	
**Parameters**	**Red**	**Green**	**Red**	**Green**	**Red**	**Green**	**Red**	**Green**
F	6	5	8	8	*7*	*5*	*8*	*8*
M	28	12	9	9	*23*	*11*	*7*	*8*
$\boldsymbol{max\{\varepsilon(R)\}}$	0.36		0.38		*0.37*		*0.28*	
$\boldsymbol{\langle\varepsilon(R)\rangle}$	0.04		0.04		*0.08*		*0.06*	

Table S1. Summary for the optimized parameters defined according to the data analysis parameters (F: the minimum photon-rate threshold and M: the minimum photon counts per burst) and protocols of Ref. 1 for the red and green fluorescence burst analyses of the four typologies of both cargo and vesicle preparations considered in the study (Ab- /dUTP cargoes and RBC/ RBC$^+$ preparations). $\boldsymbol{max\{\varepsilon(R)\}}$ and $\boldsymbol{\langle\varepsilon(R)\rangle}$ are the corresponding values of the maximum and average errors, respectively, for the relevant nanovesicle radius range (R≤100 nm), see also Ref. 1.

Based on the above analysis, the size profile of the loaded nanovesicles were evaluated and the summary of these statistics are provided in Table S2.

Sample	R_{max}^{Loaded} (nm)	R_{ave}^{Loaded} (nm)	σ_R^{Loaded} (nm)
Ab-RBC	52	66	15
Ab-RBC$^+$	52	65	15
dUTP-RBC	*(42) [1]*	*(55) [1]*	*(17) [1]*
dUTP-RBC$^+$	*48*	*61*	*17*

Table S2. Summary of the statistics on the size of loaded nanovesicles including, R_{max}^{Loaded}: the size of loaded vesicles at the peak of their distribution, R_{ave}^{Loaded}: their average size and σ_R^{Loaded}: corresponding standard deviation.

2-4- The lifetimes and brightness assessments

For calibration purposes, the fluorescence measurements were conducted on free green Alexa488-tagged Ab and dUTP cargos in PBS, under the same conditions as the main experiments. Applying the time correlated single photon counting (TCSPC) unit and data acquisition card (Hydraharp, Picoquant) in the DCFM setup, the retrieved lifetime histograms of the green channel for green-tagged cargos within RBC and RBC$^+$ nanovesicles were normalized and compared with the ones for free Ab/dUTP-Alexa488 cargo molecules in solution as shown in Figure 4 in main text and Figure S13 in next section. As apparent from the plots in Figure 4c-d and Figure S13c-d, the lifetimes of the Alexa488 dye inside the nanovesicles (RBC and RBC$^+$), were decreased with respect to the Alexa488 lifetimes of the free (non-encapsulated) Ab- and dUTP- molecules. To account for the observed lifetime shortening due to FRET interaction between Alexa488 and hemoglobulin molecules of RBC, the average count rate (brightness) corresponding to one cargo molecule inside (RBC and RBC$^+$) nanovesicles were determined by including suitable correction factors in the brightness ratios between free cargo-Alexa488 and the ones inside the nanovesicles, inferred from the experimental data. The resulting values are listed in Table S3.

**Brightness ratio**		
Cargo	RBC nanovesicles	RBC$^+$ nanovesicles
Ab-Alexa488	0.604	0.297
dUTP-Alexa488	*0.491*	*0.227*

Table S3. Correction factors for the brightness of Ab/dUTP-Alexa488 in RBC and RBC$^+$ nanovesicles, stemming from the modification of the fluorescence lifetimes resulting from the dye encapsulation inside the nanovesicles due to FRET. The values were determined from systematic comparisons of the normalized lifetime histograms, in measurements of the kind reported in Figure 4c-d at main text and Figure S11 c-d.

S3- Reference dUTP results and comparative summary with Ab-loading

The main text reports the key results concerning Ab-loading. However, the experimental procedures involved substantial further calibration results and experimental analyses using dUTP-loaded preparations, which were simultaneously processed under the same conditions as the Ab-loaded samples and used as a reference for the Ab-loading results. This section provides summaries of the main results of the corresponding reference studies performed on the dUTP-loaded nanovesicle populations (consistent with the results previously reported in Ref. 1 where the DC-CFB method was developed and applied). Figure S9 represents the size histograms of the whole population of nanovesicles measured by two separate methods including (a-b) AFM and (c-d) red fluorescence obtained from DCFM for dUTP cases in the RBC and extra cleaned RBC$^+$ preparations. The overall comparisons of these measurements along with the ones depicted in Figure 2 are provided in Table 1 at the main text.

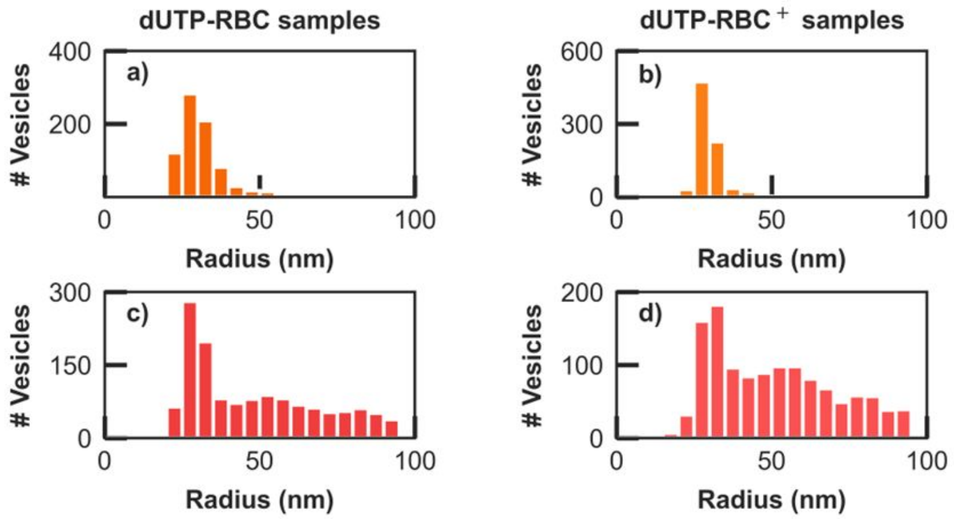

Figure S9. The size histograms of the entire (including loaded and unloaded) nanovesicles populations assessed by a-b) AFM and c-d) red fluorescence measurements (N_{tot}) in the case of dUTP cargo molecules for: (a and c) RBC, (b and d) RBC$^+$ preparations.

Based on the DC-CFB analysis, the sub-population of the loaded nanovesicles and the loading yield ($\eta(R)$) were assessed while the reference results for dUTP cases are illustrated in Figure S10.

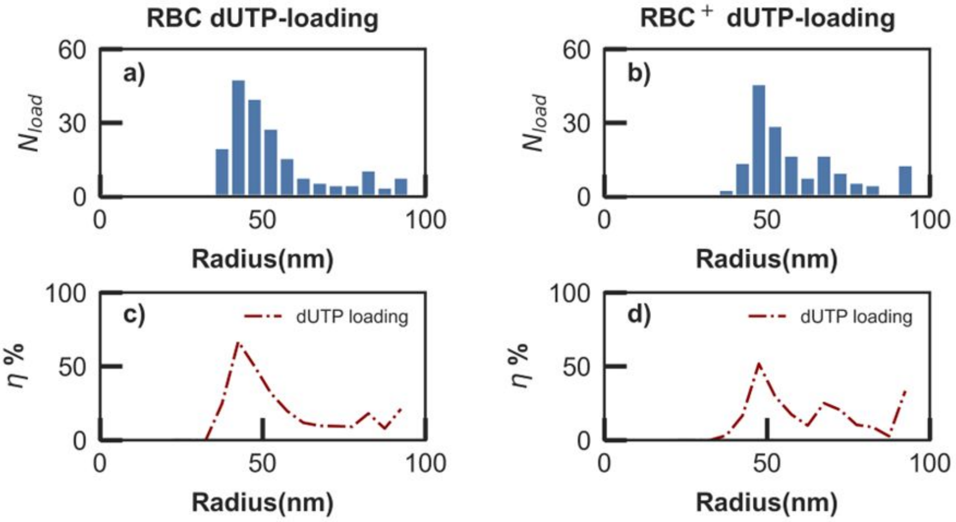

Figure S10. Size histograms retrieved from fluorescence measurements for: a-b) the loaded nanovesicle sub-populations (N_{load}) and c -d) corresponding loading yield ($\eta(R)$) profile (determined by dual color burst analysis), in the case of dUTP-loaded samples including RBC (a and c) and extra cleaned RBC$^+$ (b and d) preparations.

Moreover, after calibrating the brightness of green-tagged cargos inside the RBC and RBC$^+$ nanovesicles by comparing their lifetimes with the corresponding free cargos in solution as the brightness ratio, listed in Table S3 and shown in Figure S11 c-d.

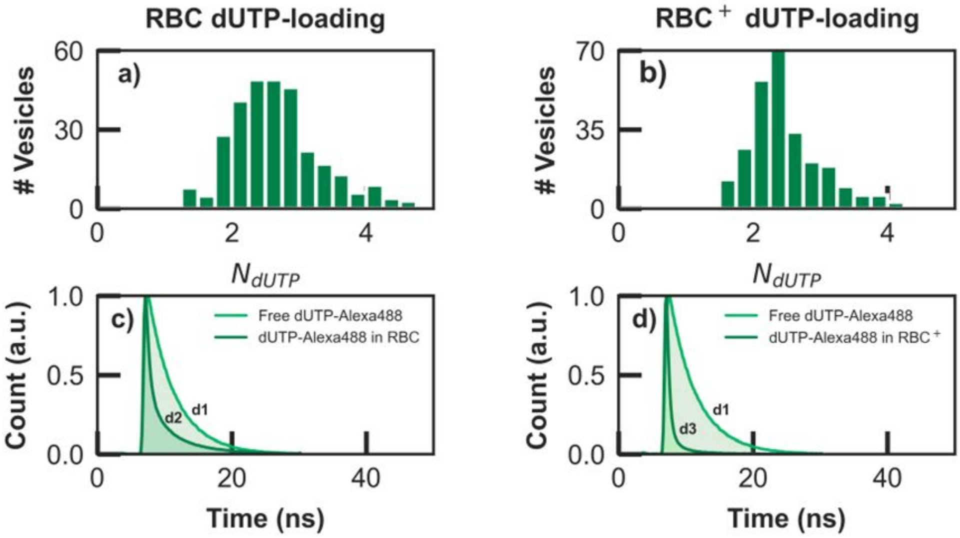

Figure S11. The histograms of the number of dUTP cargos (N_{dUTP}) loaded in (a) RBC and (b) RBC$^+$ nanovesicle inferred by comparing the normalized lifetime histograms of dUTP-Alexa488 cargos entrapped in (c) RBC (d1) and (d) RBC$^+$ (d_3) nanovesicles with the ones measured for free dUTP-Alexa488 molecule (d_1).

The histogram of the number of cargos per vesicle has been evaluated and the results for dUTP case (N_{dUTP}) are represented in Figure S11 a-b. The lifetime of the Alexa488-tagged dUTP in free solution and encapsulated inside the RBC and RBC$^+$ nanovesicles are denoted as d_1, d_2 and d_3 in Figure S11 c-d, respectively. To compare the loading yield profiles of Ab-loaded nanovesicles with dUTP-loaded ones, and RBC ones with extra cleaned RBC$^+$ ones, Figure S12 provides an overview of loading yield distributions considering both dUTP- and Ab-, loaded RBC and RBC$^+$ nanovesicles. It exhibits that Ab-loading shifted the size of nanovesicles toward slightly larger size compared to dUTP-loaded ones (Fig. S12a). Moreover, the Ab-loading yield was consistently unchanged through extra exosome spin column cleaning (Fig. S12b). However, for dUTP-loaded vesicles, the cleaning procedure had done some size filtering toward slightly larger vesicles comparable to Ab-loaded peak radius (Fig. S12c).

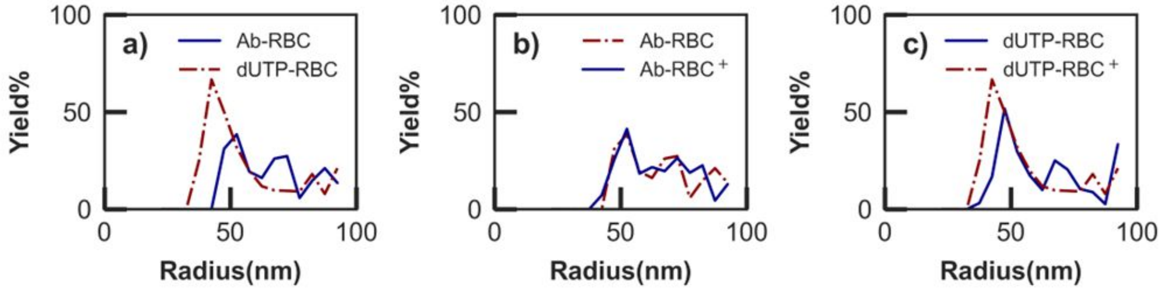

Figure S12. Comparisons between the loading yield profiles (η(R)) for: (a) Ab- versus dUTP- loaded RBC nanovesicles; (b) Ab-loaded RBC versus RBC$^+$ (extra-cleaned) nanovesicles; (c) dUTP-loaded RBC versus RBC$^+$ nanovesicles preparations.

In summary, the retrieved two-dimensional profile of loaded RBC and RBC$^+$ nanovesicles, for dUTP case, are illustrated in Figure S13, revealing the most populated size and dUTP-cargos per loaded vesicles, consistent with the results for Ab-loaded ones.

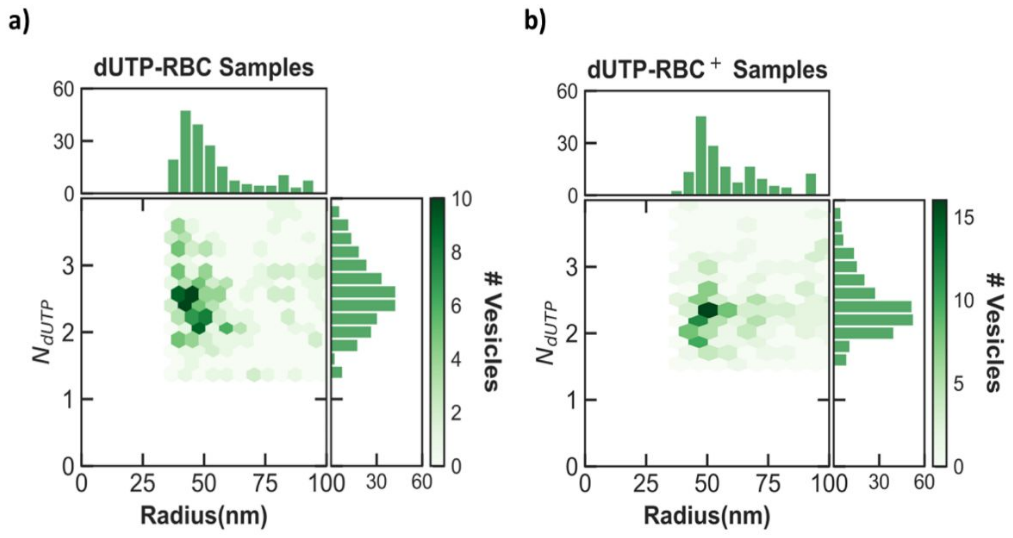

Figure S13. Two dimensional profiles of dUTP-loaded a) RBC and b) RBC+ nanovesicles, obtained from dual-color coincident fluorescence burst analysis. The horizontal and vertical subplots depict the size and number-normalized brightness per vesicle for dUTP-cargo molecules, respectively. It demonstrates that loaded nanovesicles are mostly populated at radius of 42 nm for RBC (48 nm for RBC+) and maximum number of 2.25 dUTPs per nanovesicle for both preparations (RBC and RBC$^+$).

Reference

[1] M. Sanaee, E. Sandberg, K. G. Ronquist, J. M. Morrell, J. Widengren, and K. Gallo, "Coincident Fluorescence-Burst Analysis of the Loading Yields of Exosome-Mimetic Nanovesicles with Fluorescently-Labeled Cargo Molecules," *Small,* vol. 18, no. 12, p. 2106241, 2022, doi: https://doi.org/10.1002/smll.202106241.

[2] A. M. M. Zade-Oppen, "Posthypertonic Hemolysis in Sodium Chloride Systems," *Acta Physiologica Scandinavica,* vol. 73, no. 3, pp. 341-364, 1968, doi: https://doi.org/10.1111/j.1748-1716.1968.tb04113.x.
